# MYC-mediated miR-320a affects receptor activator of nuclear factor κB ligand (RANKL)-induced osteoclast formation by regulating phosphatase and tensin homolog (PTEN)

**DOI:** 10.1080/21655979.2021.2008666

**Published:** 2021-12-21

**Authors:** Hao Chen, Shaoshuo Li, Heng Yin, Zhen Hua, Yang Shao, Jie Wei, Jianwei Wang

**Affiliations:** a Traditional Chinese Medicine Orthopedics, Nanjing University of Chinese Medicine, Nanjing, JiangSu, China; b Department of Orthopedics and Traumatology, Yancheng Dafeng Hospital of Traditional Chinese Medicine, Yancheng, Jiangsu, China; c Department of Orthopedics and Traumatology, Wuxi Affiliated Hospital of Nanjing University of Chinese Medicine, Wuxi Hospital of Traditional Chinese Medicine, Wuxi, Jiangsu,China; d PICU, Yancheng Children’s Hospital, Yancheng, Jiangsu, China

**Keywords:** MYC, miR-320a, PTEN, osteoclasts

## Abstract

Osteoporosis is a serious bone metabolism disease. Recent studies have shown that MYC could promote the formation of osteoclasts. Evidence has also shown that miR-320a could injure osteoblasts by inducing oxidative stress. By querying the database, we found that MYC has the potential to target and affect the expression of miR-320a. However, the effects of MYC and miR-320a on the the receptor activator of nuclear factor κB ligand (RANKL)-induced osteoclasts are unclear. In this study, we examined the relationship between MYC and miR-320a with luciferase reporter assay. To investigate the role of MYC and miR320a in osteoporosis, MYC or miR-320a expression were knocked down in RAW 264.7 cells. Meanwhile, the expression of markers of osteoclasts was detected with Western blotting. Finally, we inhibited the expression of PTEN in RAW 264.7 cells with miR-320a depletion and detected the expression of abovementioned proteins. MYC promoted the expression of miR-320a in RAW 264.7 cells by binding to the promoter of miR-320a. Inhibition of MYC and miR-320a suppressed the formation of RANKL-induced osteoclasts by inhibiting the expression of c-Fos, NFATc1, TRAP and CTSK. Moreover, the expression of c-Fos, NFATc1, TRAP and CTSK was rescued and the RANKL-induced osteoclasts was promoted after the repressing the expression of PTEN. In conclusion, MYC enhanced the formation of RANKL-induced osteoclasts by modulating the miR-320a/PTEN pathway.

## Introduction

Osteoporosis is characterized by reduced bone mass and degeneration of fibrous structure of bone tissue [1]. The clinical manifestation of osteoporosis is the destruction, thinning and fracture of bone trabecular structure, which leads to the increase of bone brittleness and the decrease of bone mechanical strength, and thus prone to tiny fracture or complete fracture [2]. At present, patients with osteoporosis usually relieved their symptoms by intaking the calcium and some related hormones [3,4]. Owing to the unsatisfactory therapies against osteoporosis, novel methods for the clinical treatment of osteoporosis are required to develop.

The dysfunction of osteoclast bone resorption and osteoblast bone formation will lead to osteoporosis and other bone diseases [5,6]. The function of osteoclasts is mainly to absorb bone by secreting acid and protease to dissolve the organic and mineral components of bone, thus promoting bone conversion [7]. So osteoclasts play an important role in bone remodeling. Osteoclast formation requires two key factors, namely the receptor activator of nuclear factor κB ligand (RANKL) and macrophage colony stimulating factor (M-CSF) [8]. RANKL promotes osteoclast differentiation and expression of function-related genes by activating downstream factors, ultimately leading to the generation of mature multinucleated osteoclasts [5]. Therefore, inhibiting the formation of RANKL-induced osteoclasts may be the key to the treatment of osteoporosis.

MYC, a highly efficient transcription factor, serves as a driver for the the proliferation of multiple types of cells and even the development of many malignant tumors [9]. In addition, MYC could also participate in the formation of osteoclasts in vitro. Recent study has found that MYC is a crucial transcription factor in the transcription program induced by RANKL, and its expression could also directly activate the nuclear factor of osteoclasts [10]. Furthermore, research has shown that the expression of MYC promoted the formation and differentiation of osteoclasts by modulating the expression of ERRα [11].

MicroRNA (miRNA) can regulate bone tissue metabolism by regulating osteogenic differentiation and osteoclast differentiation and maturation of mesenchymal tissue. Therefore, abnormal expression of miRNA related to bone metabolism must be closely related to the occurrence of bone metabolism related diseases such as osteoporosis [12,13]. miR-320a, recognized as a microRNA associated with the occurrence and development of osteoporosis, is found to be increased in postmenopausal women with osteoporosis [14]. Its high levels could also inhibit the activity and differentiation of mouse embryonic osteoblast precursor cells (MC3T3-E1) and promote the apoptosis of these cells [15]. In human primary osteoblasts, overexpression of miR-320a enhanced the proliferation and oxidative stress of these cells, and simultaneously suppressed the mineralization ability of these cells [16]. Therefore, we hypothesized that the expression of miR-320a could affect the formation and development of osteoclast.

Therefore, we hypothesized that the expression of miR-320a could affect the formation and development of osteoclast. In this study, our aim was to detect the role of miR-302a in RANKL-induced osteoclasts and the mechanisms involved. The results in our study could also provide the new therapy against osteoporosis.

## Material and methods

### Cell culture and transfection

The RAW 264.7 cells were obtained from the ATCC (Manassas, VA, USA). All these cells were cultured with the RPMI-1640 medium (Hyclone, USA) supplemented with 10% fetal bovine serum (Gibco, USA). They were cultured in a humidified atmosphere at 37°C with 5% CO_2_. To induce cell differentiation, cells were stimulated for five days by adding RANKL (100 ng/ml; R&D Systems, USA) to the culture medium.

miR-320a inhibitor (100 nM), negative control (NC) inhibitor (100 nM), mimic (100 nM) and NC mimic (100 nM) were synthesized by RiboBio (Guangzhou, China). miR-320a inhibitor: 5ʹ-UCGCCCUCUCAACCCAGCUUUU-3ʹ, miR-320a mimic: 5ʹ-AAAAGCUGGGUUGAGAGGGCGA-3ʹ, miR-320a mimic/inhibitor NC: 5ʹ-UUGUCCUACACCUCACUCCUG-3ʹ. Short hairpin (sh)RNA targeting mouse MYC and PTEN, as well as their specific negative control (NC) plasmids were constructed by GenePharma (Shanghai, China). When cells were fused to 80% to start the transfection procedure, cells were transfected with Lipofectamine® 2000 at 37°C for 24 h according to the manufacturer’s protocol. After 24 h, cells were rinsed with PBS and incubated with RANKL-containing medium for additional 5 days.

### TRAP staining

On the fifth day of RANKL-induced cell differentiation, cells were rinsed with PBS and fixed in 4% paraformaldehyde for 20 min. Then the cells were stained with Tartrate-resistant acid phosphatase (TRAP) stain kits (Sigma-Aldrich) according to the manufacturer’s requirements. The staining results were observed under light microscopy (Leica DM 2500; Leica Microsystems GmbH, Wetzlar, Germany), and multinucleated cells with positive TRAP staining and nuclei ≥ 3 were considered mature osteoclasts. Each group was then observed for 5 fields of view [17].

### Luciferase reporter assays

Luciferase reporter assays of MYC on miR-320a promoter fragment [18]. The miR-320a promoter fragment was inserted into the promoter region of the pGL3-basic (Promega, USA) vector. Cells (5x10^4^ cells/well) were cotransfected with sh-MYC (100 nM) or sh-NC (100 nM) with the reporter plasmid. After incubation at 37°C for 48 h, firefly and Renilla luciferase activities were measured using the Renilla-Firefly Luciferase Dual Assay kit (Pierce, Thermo Fisher Scientific, USA).

Then, the binding relationship between PTEN and miR-320a was verified. PTEN wild-type (WT) or mutant (MUT) 3ʹ-UTRs were inserted into the pMIR-REPORT vector (Ambion; Thermo Fisher Scientific, USA) expressing luciferase. The reporter plasmid (100 nM), was co-transfected with miR-320a mimic (100 nM) or NC mimic (100 nM) luciferase reporter vector (5x104 cells/well). After incubation at 37°C for 48 h, firefly and Renilla luciferase activities were measured.

### CHIP assays

RAW 264.7 cells were cross-linked with 10% formaldehyde at room temperature for 10 min and quenched with 125 mM glycine for 5 min. DNA fragments averaging 200–500 bp are produced from cross-linked chromatin using ultrasound. After that, these chromatin fragments were immunoprecipitated with Normal Rabbit IgG (CST, 2729) and MYC (Abcam, ab32072). At last, precipitated DNA were purified with the ChIP DNA Clean Kits (Zymo Research).

### qRT-PCR

Total RNA was collected with the Trizol (Thermo Fisher Scientific, USA) methods. Reverse transcription kits (Takara, Japan) were used to reverse transcribe the RNA into cDNA. After that, SYBR Green PCR kit (GeneCopoeia, USA) and the ABI7500 system (Thermo Fisher Scientific, USA) were used for the amplified of the cDNA. The conditions were as follows: 95°C for 30 sec, followed by 40 cycles of 95°C for 10 sec and 60°C for 30 sec. And the results were analyzed with the 2^−ΔΔCt^ method.

### Western blotting

Total protein was extracted with the RIPA buffer (Beyotime, China). Then, the concentration of these samples was determined with the BCA method (Beyotime, China). After that, these proteins were separated with the 8%-12% SDS-PAGE gel (Beyotime, China). These proteins were then transferred to the PVDF membranes (Millipore, USA). Then, these membranes were blocked with 5% skim milk powder. Following that, these membranes were incubated with primary antibodies at 4°C overnight. The primary antibodies used in this study were MYC (1:500; Abcam, ab32072), c-Fos (1:500; Abcam, ab222699), nuclear factor of activated T cell (NFATc1, 1:500; Abcam, ab25916), tartrate-resistant acid phosphatase (TRAP, 1:600; Abcam, ab52750), cathepsin K (CTSK, 1:600, Abcam, ab37259), PTEN (1:600, Abcam, ab267787) and GAPDH (1:1000, Abcam, ab9485). These membranes were incubated with the horseradish peroxidase-conjugated secondary antibody (1:1000; Cell signaling technology, 7074) for 2 h on the second day. Finally, the immunoreactive signals were detected by the Pierce Western blotting Substrate (Thermo Fisher Scientific, USA).

### Statistical analysis

All the data in this study were analyzed with the GraphpadPrism 6.0 and displayed as mean ± SD. All the experiments in this research were repeated for three times. The comparison between diverse groups was performed with the student’s t test. The difference was considered as statistically significant when the value of P was less than 0.05. The study was allowed to be repeated by other Authors.

## Results

### The expression of miR-320a was upregulated in RANKL induced osteoclast

**Figure 1.**
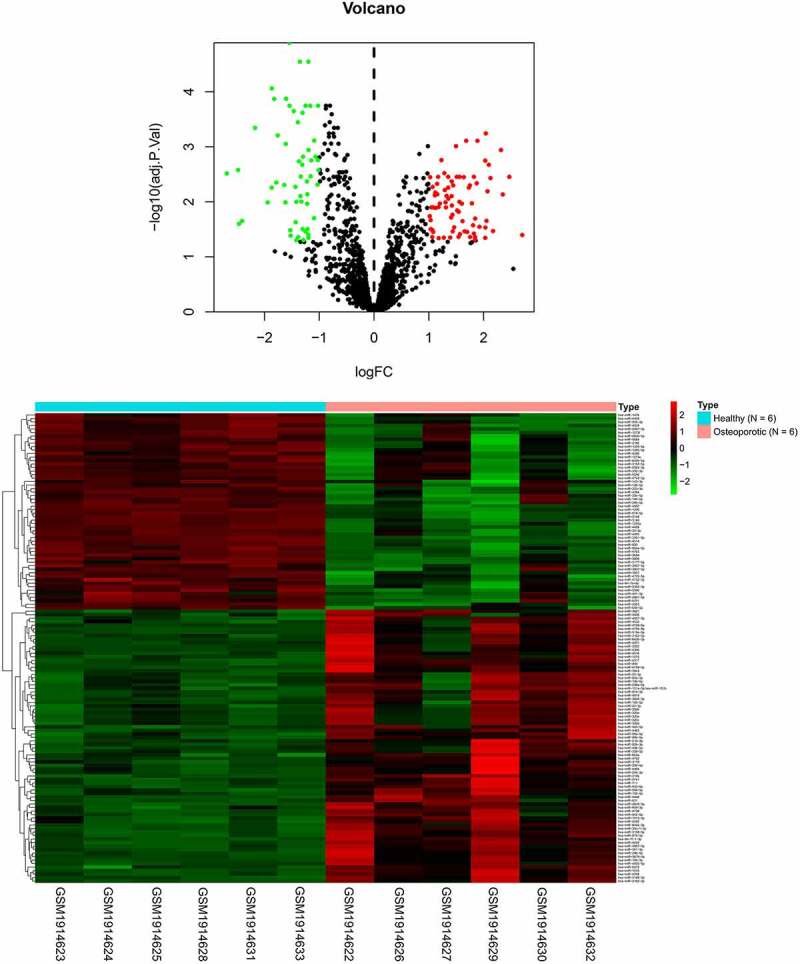
The expression of related miRNAs in patients with osteoporosis. (a) The expression of miRNAs in osteoporosis patients and normal people. (b) Gene chip was used for the detection of the difference of the expression of miRNAs between osteoporosis patients and normal people

**Figure 2.**
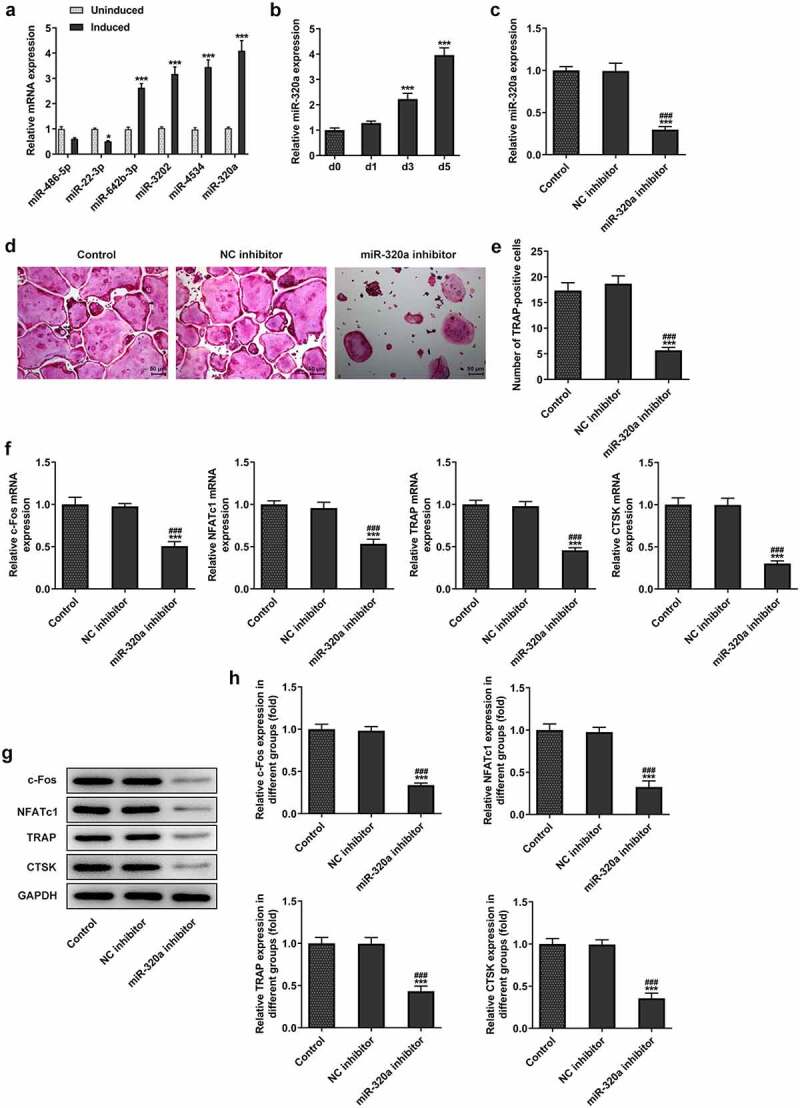
Inhibition of miR-320a suppressed the formation of RNAKL induced osteoclasts. (a) RT-PCR was performed to detect the expression of miRNAs during the formation of osteoclasts. (b) RT-PCR was performed to determine the expression of miR-320a in the RNAKL induced osteoclasts. (c) RT-PCR was carried out to detect the expression of miR-320a in miR-320a knockdown osteoclasts. (d) and (e) TRAP staining was used for the detection of the osteoclasts. (f) and (g and h) The expression of markers of osteoclasts was detected with the q-PCR and Western blotting. *p < 0.05, **p < 0.01, ***p < 0.001

By querying the database (GEO, GSE74209, https://www.ncbi.nlm.nih.gov/geo/), we found that the expression of 56 miRNAs was downregulated and the levels of 78 miRNA were increased in the tissue of osteoarthritis patients (Figure 1a and Figure 1b). Subsequently, we examined the expression of several miRNAs, including miR-486-5p, miR-22-3p, miR-642b-3p, miR-3202, miR-4534 and miR-320a. It showed that the most significant upregulations during RANKL-induced osteoblast differentiation. The results (Figure 2a) showed that the expression of miR-320a was increased most significantly during the development of osteoclasts. As shown in Figure 2b, the expression of miR-320a was enhanced during the RANKL-induced osteoclasts. Next, we knocked down miR-320a in osteoclasts to further determine the effect of miR-320a on the development of osteoclasts. The results (Figure 2c) from q-PCR showed that the expression of miR-320a was decreased in the cells of knockdown group. The expression of c-Fos, NFATc1, TRAP and CTSK was the symbol of the osteoclasts [19]. Therefore, we detected the expression of these proteins in miR-320a knockdown cells. Results (Figure 2d and 2e) from TRAP staining showed that the TRAP-positive osteoclasts were decreased after the knockdown of miR-320a. In addition, the expression of c-Fos, NFATc1, TRAP and CTSK was suppressed after the inhibition of miR-320a in these cells (figure 2f, 2g and 2h).

### MYC promoted the expression of miR-320a in osteoclasts

**Figure 3.**
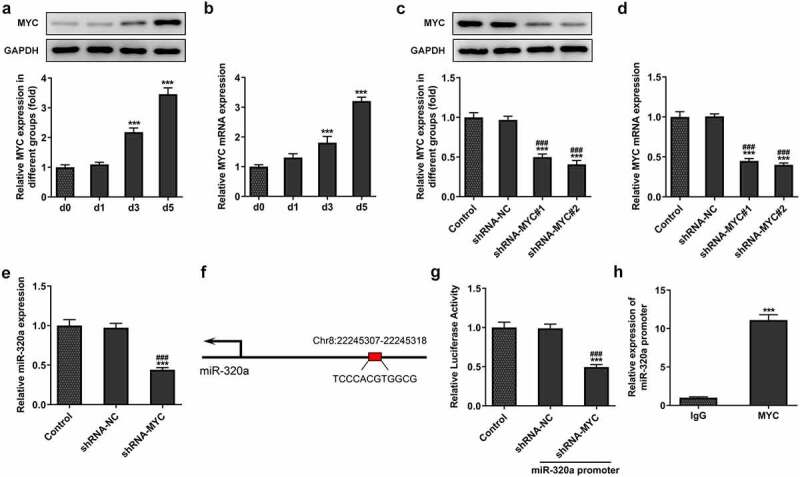
MYC promoted the expression of miR-320a in osteoclasts. (a) and (b) The expression of MYC in osteoclasts was detected with the Western blotting and q-PCR. (c) and (d) Levels of MYC in MYC knockdown osteoclasts were detected with the Western blotting and q-PCR. (e) The expression of miR-320a in MYC inhibition osteoclasts was determined with the q-PCR. (f) The potential binding sites between MYC and miR-320a. (g) Luciferase reporter assays were performed to detect the relationship between MYC and miR-320a. (h) CHIP assays were used for the verification of the association between MYC and miR-320a. *p < 0.05, **p < 0.01, ***p < 0.001

For the detection of the effect of MYC on the formation and development of osteoclasts, we determined the expression of MYC in RANKL-induced osteoclasts. The results showed that the expression of MYC was increased during the proliferation of osteoclasts (Figure 3a and 3b). Next, by establishing the MYC knockdown osteoclasts with the transfection, the expression of MYC was repressed in the cells of knockdown group (Figure 3c and 3d). Furthermore, the expression of miR-320a also decreased after the knockdown of MYC in these cells (Figure 3e). By querying the database (JASPAR, http://jaspar.genereg.net/), we found that the MYC has the potential to bind to and regulate the expression of miR-320a (figure 3f). The results (Figure 3g) from luciferase reporter assay also showed that the fluorescence intensity was decreased in the miR-320a promoter and sh-MYC system. In addition, CHIP assays also performed to detect the relationship between miR-320a and MYC. The CHIP result (Figure 3h) showed that MYC bound to the promoter region of miR-320a.

### miR-320a targeted and suppressed the expression of PTEN

**Figure 4.**
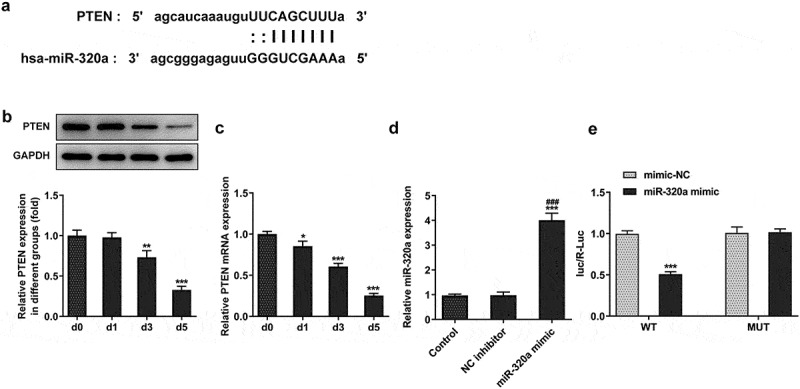
miR-320a targeted the PTEN in osteoclasts. (a) The potential binding sites between miR-320a and PTEN. (b) and (c) The expression of PTEN was detected with Western blotting and q-PCR in RNAKL induced osteoclasts. (d) The expression of miR-320a in miR-320a overexpression osteoclasts was detected with q-PCR. (e) Luciferase reporter assays were performed to detect the relationship between miR-320a and PTEN. *p < 0.05, **p < 0.01, ***p < 0.001

The data from the database (ENCORI, http://starbase.sysu.edu.cn/) showed that miR-320a has the potential to regulate the expression of phosphatase and tensin homolog (PTEN) (Figure 4a). The expression of PTEN suppressed the formation of osteoclasts [20]. The results from our assays also showed that the expression of PTEN was decreased in the RANKL-induced osteoclasts (Figure 4b and 4c). Then, we established the miR-320a overexpression osteoclasts and the results (Figure 4d) showed that the levels of miR-320a in cells of overexpression groups were upregulated. Results (Figure 4e) from luciferase reporter assay also showed that the fluorescence intensity was reduced in the PTEN wild type and miR-320a overexpression system.

### miR-320a promoted the formation of osteoclasts by inhibiting the expression of PTEN

**Figure 5.**
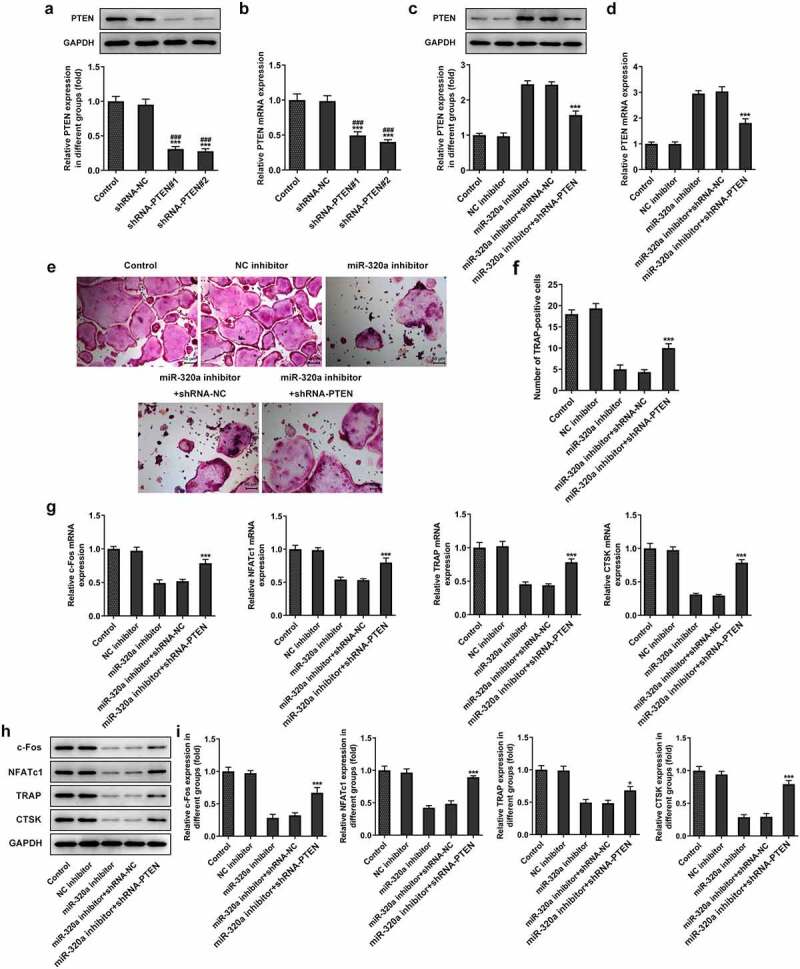
miR-320a promoted the formation of osteoclasts by inhibiting the expression of PTEN. (a) and (b) The expression of PTEN in PTEN knockdown osteoclasts was detected with Western blotting and q-PCR. (c) and (d) Western blotting and q-PCR were performed to detect the expression of PTEN in osteoclasts. (e) and (f) TRAP staining was used for the detection of the osteoclasts. (g) and (h and i) The expression of markers of osteoclasts was detected with the q-PCR and Western blotting. *p < 0.05, **p < 0.01, ***p < 0.001

For the detection of the effect of PTEN on the formation of osteoclasts, we established the PTEN knockdown osteoclasts. The results (Figure 5a and 5b) showed that the expression of PTEN in knockdown groups was decreased. Because PTEN shRNA-2 has the better knockdown effect, we selected PTEN shRNA-2 for the subsequent experiments. Next, we detected the expression of PTEN in miR-320a inhibitor osteoclasts. As shown in Figure 5c and 5d, the levels of PTEN were increased in miR-320a inhibition osteoclasts. The expression of PTEN was repressed after the knockdown of PTEN. In addition, the staining of TRAP also showed that the number of TRAP-positive cells was decreased after the inhibition of miR-320a, which was rescued after the inhibition of PTEN (Figure 5e and 5f). Similarly, the expression of c-Fos, NFATc1, TRAP and CTSK was also suppressed after the knockdown of miR-320a. The restriction of PTEN also rescued the expression of these proteins in osteoclasts (Figure 5g, 5h and 5i).

## Discussion

Osteoporosis is a common metabolic bone disease characterized by bone loss and structural destruction [21]. The gradually increased incidence of osteoporosis seen by recent years has called for a new therapy against this disease [22]. The bone mass homeostasis in many bodies gradually declined at the middle ages of people, which could lead to bone loss, osteoporosis, and even debilitating fractures [23]. Osteoblasts played a critical role in the process of bone formation and differentiation [24]. In the presence of osteoblasts, the wild development of osteoporosis could be blocked through regulating the bone synthesis [25].Moreover, osteoclasts could absorb damaged bone and convert it into raw material for new bone formation, which directly contribute to the bone development [26]. Therefore, the formation of osteoclasts is also the critical process during the growth of the bone.

MYC, a transcription factor with a wide range of functions, could regulate cell differentiation and proliferation through a variety of mechanisms [27,28]. Recent study has indicated the importance of MYC in modulating the bone metabolic process. Study revealed that the expression of MYC promoted the formation of osteoclasts in vitro and regulated bone loss by activating the ERRα in physiological and pathological means [11]. In our study, we confirmed that the expression of MYC promoted the formation of osteoclasts. In addition, miR-320a, a microRNA associated with the growth and development of osteoblasts, has been indicated to relieve the symptoms of osteoporosis by suppressing the Wnt/β-catenin pathway, in the company of lncRNA DANCR [14]. However, study suggested that the expression of miR-320a, which was promoted in postmenopausal osteoporosis, could induce the oxidative damage of osteoblasts by suppressing the PI3K/AKT signaling pathway [15,16]. In our study, we found that MYC has the potential to target and regulate the expression of miR-320a. Mechanistically, the expression of MYC enhanced the expression of miR-320a by binding to the promoter region of miR-320a. NFATc1 and cFos are critical transcription factors that promoted the formation of osteoclasts [29]. The expression of TRAP and CTSK also played the critical role during the formation of osteoclasts [30]. The results in this study indicated that the expression of MYC also promoted the formation and development of RANKL-induced osteoclasts by promoting the expression of cFos, NFATc1, TRAP and CTSK.

In addition, PTEN is a multifunctional molecule that exists in multiple cells. The expression of PTEN could modulate various cellular processes such as cell proliferation, survival, adhesion, movement and apoptosis [31,32]. Besides its role in influencing cellular processes, its main function is to negatively regulate many signal pathways, such as PI3K/AKT and NF-κB pathway [33]. Interestingly, recent study has found that PTEN is an important regulator during the process of osteoclast formation-induced by RANKL, associating the occurrence of osteoclast formation with PTEN [34]. Specifically, PTEN inhibited RANKL-induced osteoclast formation in RAW 264.7 cells by negatively regulating the AKT and NF-κB signaling pathways induced by RANKL [35]. In our study, we also found that the inhibition of miR-320a suppressed the formation of RANKL-induced osteoclasts by promoting the expression of PTEN. The knockdown of PTEN relieved the suppression of the formation of RANKL-induced osteoclasts. Therefore, these results indicated that the expression of miR-320a promoted the RANKL-induced osteoclasts by suppressing the expression of PTEN.

Our article also has limitations. We only tested it in cells, but not in vivo in animals. In addition, in the experiment, we only discussed the up-regulation of PTEN expression by miR-320a mediated by MYC, but did not detect the influence of overexpression or knockdown of MYC on PTEN expression. We will explore those further in future experiments.

## Conclusion

Above all, we clarified the effect of MYC/miR-320a/PTEN on the RANKL-induced osteoclasts in this study. The results in our study also implied that the MYC enhanced the RANKL-induced osteoclasts by promoting the expression of miR-320a and inhibiting the expression of PTEN. The conclusion of our study could also provide a new therapy against osteoporosis.

## Data Availability

The datasets used and/or analyzed during the current study are available from the corresponding author on reasonable request.

## Research highlights

- MYC promoted the expression of miR-320a in osteoclasts
- miR-320a targeted and suppressed the expression of PTEN in osteoclasts
- miR-320a affects RANKL-induced osteoclast formation
